# Team-Based Analysis of Large-Scale Qualitative Data: Tutorial Using a Nationwide SMS Text Messaging Poll of Youth

**DOI:** 10.2196/72526

**Published:** 2026-02-27

**Authors:** Melissa DeJonckheere, Samantha A Chuisano, Marika Waselewski, Kendrin Sonneville, Tammy Chang

**Affiliations:** 1 Department of Family Medicine University of Michigan Ann Arbor, MI United States; 2 Institute for Healthcare Policy and Innovation University of Michigan Ann Arbor, MI United States; 3 School of Public Health, Department of Nutritional Sciences University of Michigan Ann Arbor, MI United States

**Keywords:** qualitative, large-scale, survey methods, data analysis, adolescence

## Abstract

With the growing use of technology in qualitative data collection and analysis, there is an opportunity to gather rich and varied perspectives to improve health and well-being. However, large-scale qualitative datasets can be difficult to manage using traditional qualitative methods, and there are few examples of the application of large-scale qualitative analysis. In the context of digital health, large qualitative datasets are increasingly made up of short text segments, which need to be analyzed differently from lengthy transcripts from interviews or focus groups. Therefore, this tutorial describes the use of traditional qualitative methods to analyze a large corpus of qualitative text data. We use examples from a nationwide SMS text messaging poll of youth to highlight the opportunities to use this team-based analysis approach, which has been accessible and meaningful to youth researchers and novice qualitative researchers. These large-scale qualitative strategies may benefit novice researchers analyzing large volumes of qualitative data and short text segments, including SMS text messaging, social media posts, medical notes, and open-ended survey questions, among others.

**International Registered Report Identifier (IRRID):**

RR2-10.2196/resprot.8502

## Introduction

Large-scale qualitative methods can gather the diverse perspectives of many people to augment our understanding of complex health and social issues. Traditional qualitative methods have focused on depth rather than breadth of analysis, resulting in smaller samples with rich detail. However, large-scale qualitative methods can emphasize both depth and breadth, responding to common critiques of qualitative research related to external validity (ie, generalizability in quantitative research or transferability in qualitative research). Large-scale qualitative approaches are appropriate for some studies and research questions, including those that use large primary or secondary text data sources (eg, social media posts, documents, and medical records) [1], multiple sites for data collection [2], longitudinal studies [3], or mixed-methods studies where understanding experiences across all participants is desirable (eg, clinical trials) [4-6]. Advances in technology have also increased access to large qualitative data sources through data mining techniques, such as social media sites, electronic health records [7,8], and patient portal messages [4]. As a result, mobile health and other digital health researchers have access to vast qualitative datasets of short text data.

Much of the existing literature on large-scale qualitative studies has focused on collaborative research teams that collect data across multiple sites, institutions, states, or nations. Traditional approaches for qualitative analysis leverage a line-by-line review of each interview transcript or open-ended text response. However, the time and resources required to apply these approaches to a dataset with thousands of qualitative datapoints would be restrictive for nearly all research teams. Little practical guidance exists for researchers on how to approach data management and analysis of these large datasets [6,9]. As a result, some teams may intentionally narrow their analytic focus using purposive sampling techniques [3,5], including a subsample of responses into their analysis, or use rapid approaches to qualitative analysis [10,11]. Furthermore, many teams leverage software to assist with data management, coding, or summarization [12-14]. However, these approaches may introduce the need for additional time and resources for learning and acquiring new tools or new methodologies [13,15].

The growth of artificial intelligence (AI) has shaped some approaches to qualitative data analysis as some researchers seek methods to improve efficiency or increase objectivity [16]. For example, natural language processing has been used to support text analysis of large text datasets through the initial development of a coding structure or validation of themes [4,17,18]. Large language models, like ChatGPT (OpenAI), have been used to explore the use of qualitative analysis through summarization and theme development [19-21]. However, AI-supported qualitative analysis has thus far been limited in its ability to understand nuance and context in qualitative data and develop interpretive themes [18,21]. Furthermore, some qualitative researchers have rejected the use of AI in interpretive and reflexive qualitative approaches, which are intentionally human and subjective processes used to make meaning of human thoughts and experiences [22].

As human interpretation is still integral to qualitative research, practical guidance for researchers seeking to conduct analysis of increasingly large and complex studies in digital health is needed [6,9]. While analysis can be burdensome, human-based qualitative processes can be conducted efficiently on large datasets without sacrificing methodological rigor. Likewise, analysis of large qualitative datasets can be completed without the need for advanced methodologies or acquisition of new software [15]. Therefore, the purpose of this tutorial is to describe an example of team-based qualitative analysis of a large-scale SMS text message poll using traditional qualitative methods and freely available, easy to learn collaboration tools, including cloud-based spreadsheets. While spreadsheets and matrices have been used to organize and analyze transcripts from interviews and focus groups, [23,24] there is limited guidance on strategies for datasets with a large volume of short text responses common in digital health studies. We describe our experience conducting team-based qualitative analysis of open-ended survey responses from a nationwide SMS text messaging poll of youth across the United States [25]. Our approach has been used to efficiently and effectively engage both new and experienced researchers in qualitative research, including youth, students, and trainees.

## Methods

### Example Study

To illustrate our analysis approach, we use MyVoice, a nationwide poll of individuals aged 14 to 24 years across the United States that is administered via SMS text messaging [25]. The goal of MyVoice is to gather diverse youth perspectives across the United States to inform policies and programs that impact youth. Incorporating youth voices can create more relevant, effective, sustainable, and impactful interventions that ultimately improve the health of young people [26,27]. Increasingly, research has included the perspectives of adolescents, rather than adult proxies, while also considering the unique needs of this age group [28]. Participatory approaches to research have also successfully integrated adolescents into the research team to increase the relevance of research to youth populations while also promoting youth development and capacity [29-31]. However, engaging adolescents in research remains challenging when conducted in traditional settings and when using methods that are not accessible or approachable to young people.

MyVoice is designed to address challenges related to engaging youth in research through familiar and easy-to-use methods, like SMS text messaging to collect personal opinions and stories. Each MyVoice poll consists of 4-5 primarily open-ended questions designed to elicit youth perspectives on complex health and social issues. Participants respond to questions directly via SMS text messaging, rather than following a link to an external platform. The MyVoice research protocol was previously described after enrolling approximately 100 participants [25]. While the study rationale and overall methodology have not changed since 2016, there are currently approximately 800 MyVoice participants in each cohort to maximize understanding of variations in their lived experiences to better inform policy and practice. A brief description of the overall methods is presented below, highlighting considerations for large-scale data collection relevant to this tutorial. For additional details on recruitment, screening, and enrollment of MyVoice participants, see Multimedia Appendix 1.

### Weekly Polls

SMS text messaging polls are administered using a cloud-based platform to send and receive SMS messages directly, which includes Textizen and the University of Michigan instance of REDCap (Research Electronic Data Capture; Vanderbilt University) with Twilio integration. REDCap is a secure, web-based software platform designed to support validated data capture, data management, and integration for research studies [32,33]. Questions are sent one at a time, and respondents must send an SMS text message to receive the next question. Participants are given 7 days to complete the poll before the next poll is automatically sent. Participants who have not responded after 2-3 days are sent a reminder to complete the poll. Each week, approximately 2750-3250 open-ended responses are collected. Since 2016, we have collected over 120 SMS text messaging polls and 45,000 unique responses.

### Ethical Considerations

The MyVoice study was approved by the Institutional Review Board at the University of Michigan Medical School (HUM00119982). All participants provide online informed consent or assent (for those under the age of 18 years) prior to enrollment in the study. Parental consent is waived for minors. Participants receive US $1 for each poll that they answer (each poll consists of 4-5 questions) and US $5 for completion of a demographic survey upon enrollment. All study data are collected and stored in secure web-based platforms. Data are deidentified before shared with team members and any external collaborators.

### Data Analysis of Large-Scale Qualitative Data

#### Analysis Overview

Below, we outline the efficient and reliable method we have used for team-based coding of large qualitative datasets. The overall approach includes five distinct steps: (1) data cleaning and management, (2) codebook development, (3) coding, (4) reconciliation, and (5) presentation of findings, which are described in detail below. Our approach leverages traditional qualitative approaches (ie, manual human coding) and enables collaboration among teams at various experience levels by using a simple spreadsheet – a freely accessible tool that can be used with minimal to no training. While spreadsheets have been used to structure coding of interview and focus group data, few studies [15,23,24,34] have described the use of spreadsheets for qualitative analysis of short open-ended text responses. Worked examples can be particularly beneficial in highlighting strategies that have been successfully implemented by other researchers to manage qualitative data and make study design decisions [15,35]. In the example provided, we highlight specific considerations for large-scale short text responses from the MyVoice study; however, this method could be easily applied to any study where data are available in a table or a .csv format (ie, social media data, unstructured medical record text, open-ended survey responses, etc).

#### Step 1: Data Cleaning and Management

SMS text messaging responses are downloaded from the data collection platform as a .csv file. Data are deidentified when downloaded and identified by a participant identification number. Cleaning includes merging responses with participant demographic data, rearranging lines of text that were separated, preliminary identification of junk or skipped responses, and filtering out nonresponses (ie, when a respondent does not answer a question).

The resulting deidentified spreadsheet is organized with the following columns: participant study ID, responses separated by question, and demographic survey responses, each in a separate column. Each row in the file includes the data for a unique participant.

For all MyVoice studies, analyses include line-by-line coding of deidentified participant responses in a spreadsheet. Teams primarily use a cloud-based platform (eg, Google Sheets) to ensure that all members of the research team (including high school students, undergraduate students, and other trainees) have access to the data throughout the analysis process and are able to work synchronously and asynchronously.

#### Step 2: Codebook Development

Codebooks are used to systematically record and organize qualitative data analysis by providing a list of key ideas in a dataset (ie, codes) and ensuring consistency and transparency in analysis [13]. MyVoice teams typically use a structured codebook to record the emerging ideas in their large-scale text data, including codes, definitions, and examples [13]. The codebook development process includes three overarching and iterative steps: (1) data familiarization through open coding and memoing, (2) identifying shared concepts across team members, and (3) defining and refining codes. Our codebooks are developed iteratively and collaboratively, with multiple team members offering interpretations of the important ideas that are present in the dataset and revising the list of codes throughout the analysis process [36].

#### Data Familiarization

Within each study team, at least 2 members independently read a portion of participant responses. Team members read approximately 100 responses per question, which in most datasets is approximately 10-20% of the responses. Multiple research team members independently use open coding to list the main concepts in the responses in an analytic memo, a document reflecting the researcher’s thinking throughout the analysis process [13,37]. In our team, memos typically include a running list of concepts that are repeated in the data that will inform future codes. Some researchers include personal reflections, emerging patterns, and connections to the literature in their analytic memos [13].

#### Identifying Shared Concepts

Team members compare analytic memos to create a synthesized list of concepts that were identified in the responses. These concepts form the basis of the codes, although they may be renamed or modified in subsequent steps.

#### Defining and Refining Codes

Each concept is defined in a structured codebook that includes the code (ie, label for the concept), definition, and example response [13,38]. Some teams also include inclusion and exclusion criteria in their codebook, depending on the type of question and desired overlap between codes [38]. In studies with poll questions that are close-ended or when using a more deductive analysis approach, responses are typically categorized into discrete codes with clear inclusion and exclusion criteria. Teams may also structure their codebook with rules for co-coding responses, which can be helpful when analyzing responses that include both categorical and narrative responses.

**Figure 1:**
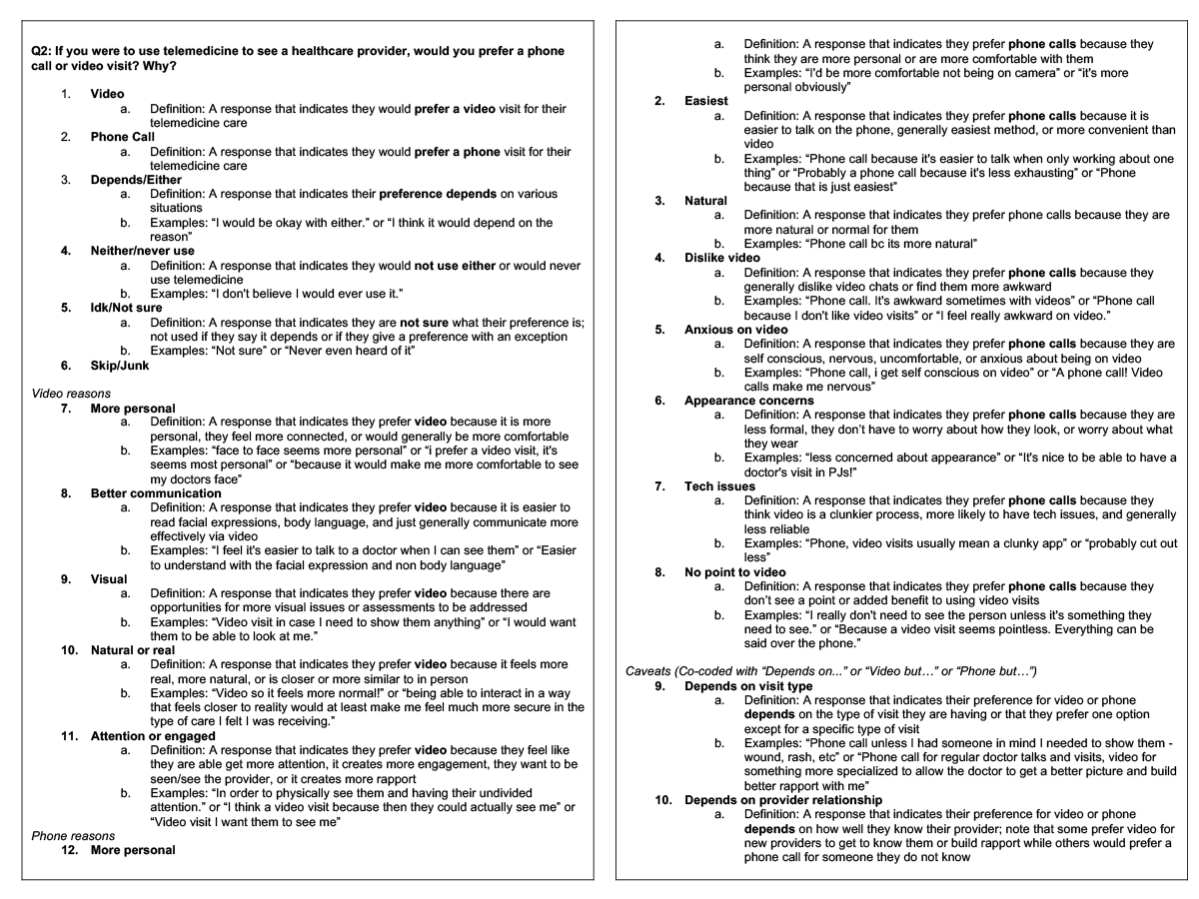
Excerpt from structured codebook organized by poll question (Waselewski et al, 2022 [39]).

Figure 1 displays an excerpt from a structured codebook that was used to analyze a dataset on youth perspectives on telemedicine before and after the start of the COVID-19 pandemic [39]. In total, 5 open-ended questions were asked in October 2019, and a new set of 5 questions was asked in October 2020. A separate coding scheme was created for all 10 questions.

For example, in the October 2020 poll, 1 question read, “If you were to use telemedicine to see a health care provider, would you prefer a phone call or video visit? Why?” The research team inductively developed codes for this question that included categorical codes that captured the first part of the question (ie, preference for phone or video visits) and descriptive codes for the subsequent explanation. Categorical codes included “Video,” “Phone call,” “Depends or either,” “Neither or never use,” or “I don’t know or not sure” to understand participants’ preferences for video or phone visits when using telemedicine. Here, there is a clear distinction in the categories, and responses cannot be categorized into multiple codes.

Each categorical code was also accompanied by a list of reasons that were derived from the text responses explaining their preference between phone and video visits (ie, descriptive codes). As seen in Figure 1, respondents who indicated a preference for telemedicine using video often indicated in their responses that video visits were more personal, improved communication, included visuals that could help with assessment, better approximated an in-person visit, and improved engagement with the provider. These reasons were then translated into descriptive codes that could be co-coded alongside “Video” (eg, “more personal,” and “better communication”). Respondents who preferred phone visits described them as more personal, easy, and natural, among other factors. These reasons were translated into descriptive codes that could only be co-coded with “Phone call.” While this team had rules for co-coding such that the concept of “more personal” had a code for preferring video visits and a separate code for preferring phone calls, an alternative strategy would be to have a single code of “more personal” that could be applied across categorical codes. Decisions are made by the research team to determine the scope of their codebook and direction for analysis. In this example, having separate codes allowed for quick calculations of frequency for each code in the content analysis.

Most MyVoice teams create a separate coding scheme for each question (as in Figure 1). A separate codebook for each question is helpful when conducting a content analysis, when the questions and, therefore, responses vary significantly, or to focus the analysis. It is also possible to create an overarching coding scheme that can be applied across questions in a single dataset. A single, overarching coding scheme would be appropriate when the poll questions are closely tied, and content from multiple responses can be captured with related codes. Alternatively, if analyzing all the data by individual respondent, rather than by question, a single coding scheme would also be optimal. This approach would closely align with most thematic analysis approaches that proceed with line-by-line coding using an individual participant or individual interview transcript, observation, or other textual data as the unit of analysis.

**Figure 2:**
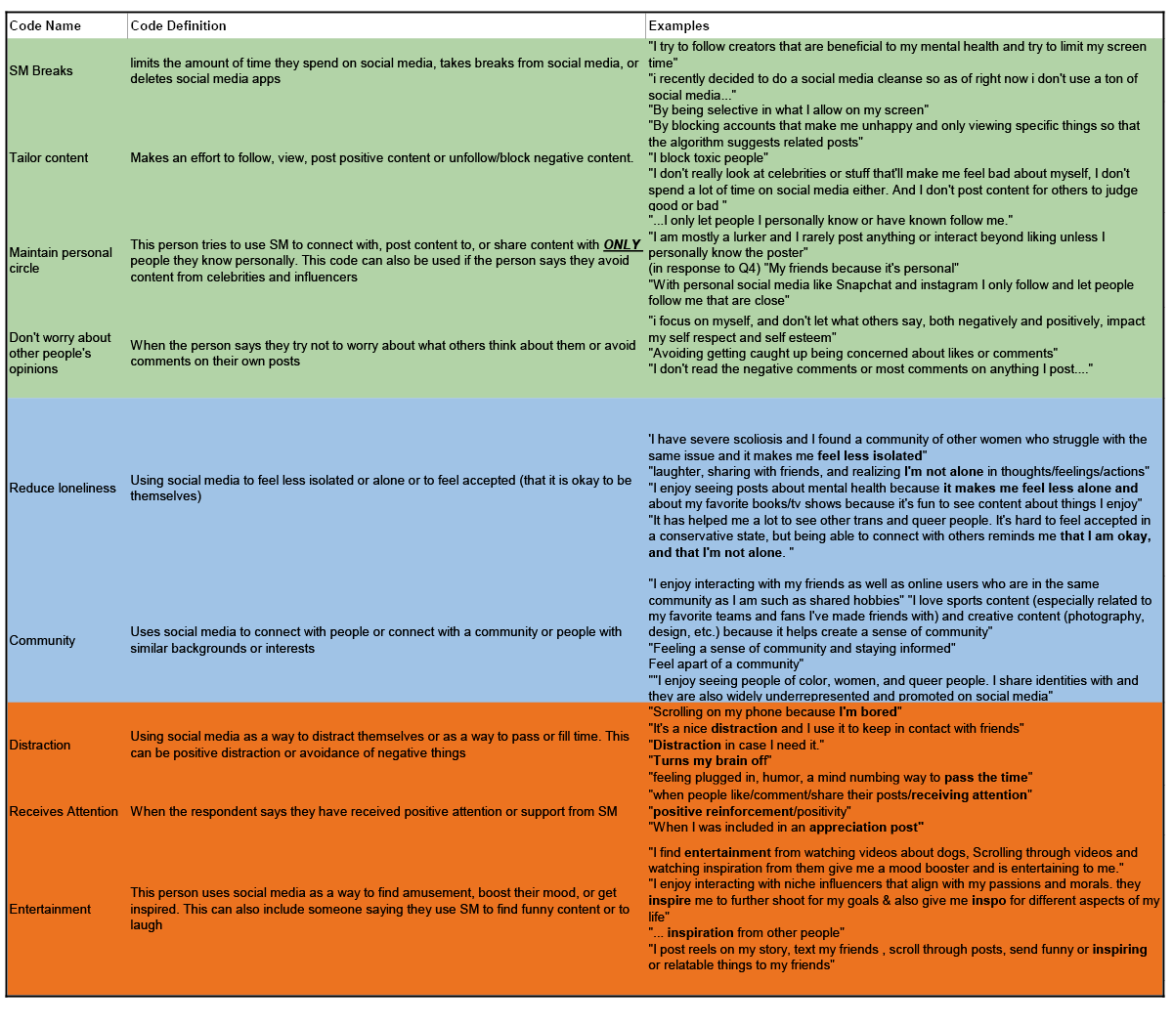
Example excerpt from MYHealth social media dataset with single codebook.

See, for example, an excerpt from a codebook developed by high school student co-researchers [40]. During the MYHealth research training program, high school students developed a study to understand youth perspectives on the impact of social media on adolescents and young adults. Poll questions included the following: (1) How do you typically use social media (posting, watching videos, scrolling, messaging, commenting, etc.)? (2) What benefits do you get from social media? (3) How do you create a positive experience for yourself while using social media? (4) Who or what do you enjoy seeing or interacting with on social media? Why? (5) Tell us about a time when social media made you feel good about yourself. This team elected to analyze responses across individual participants, rather than by question, to track patterns across all 5 questions. See Figure 2 for an excerpt from the resulting codebook.

#### Step 3: Coding

In Google Sheets, Microsoft Excel, or another spreadsheet, each code from the finalized codebook is entered into a new, separate column. Within each research team, at least 2 members independently perform line-by-line coding of each participant's response. Cloud-based tools allow team members to collaboratively code data in real-time.

Team members use the codebook developed in the previous step to apply codes to each participant's response. Where a concept is described by the participant, a “1” is entered into the spreadsheet cell. All other cells remain blank (or could be entered as “0”). In most cases, multiple codes may be applied to a participant’s response to reflect the many ideas that are present. Many responses include multiple sentences, phrases, or lists of ideas that are unique concepts.

For example, in response to the question about preference between video or phone visits for telemedicine, one respondent shared:

Video visit, for sure. Because the non-verbal cues that come along with being able to see someone are extremely important. And with my primary, I wouldn’t feel satisfied with a phone call because being able to interact in a way that feels closer to reality would at least make me feel more secure in the type of care I was receiving.

**Figure 3:**
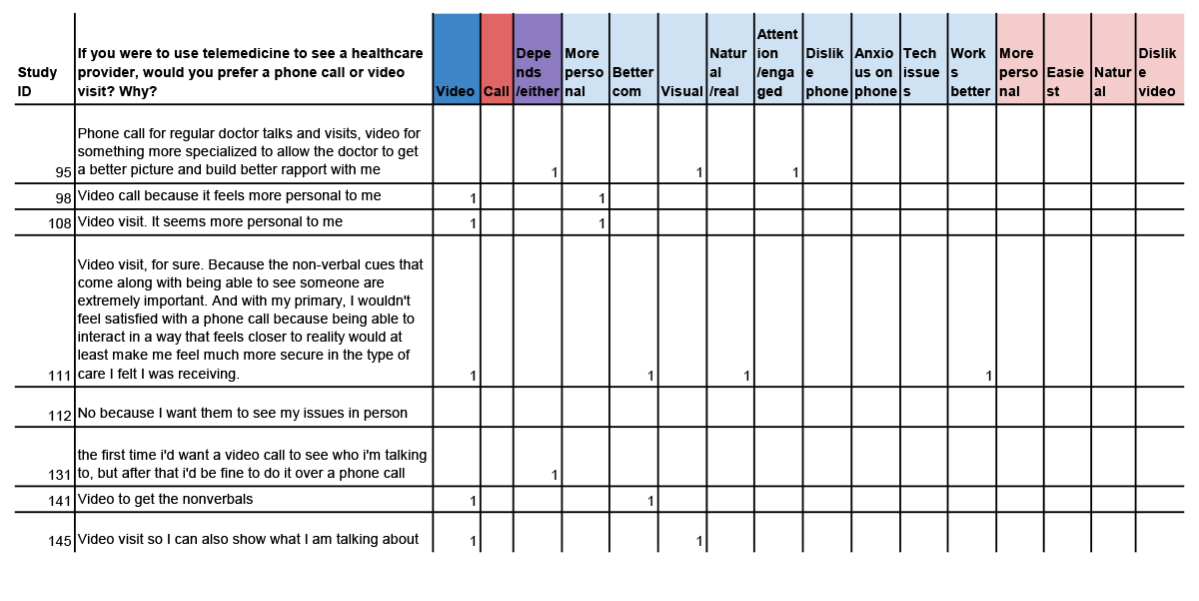
Example coding sheet and completed coding.

This response included several important concepts, which should each be coded to accurately reflect the data. The research team identified the following codes: (1) video, (2) better communication, (3) natural or real, and (4) works better (represented in the highlighted row in Figure 3).

#### Step 4: Reconciliation

Depending on the analysis strategy, teams often review and reconcile discrepancies in coding to reach agreement on the meaning of each response and final assignment of codes. Teams will use a spreadsheet formula to quickly identify discrepancies in applied codes across the large dataset. Disagreements are labeled in a new column so they can easily be filtered and reviewed. To resolve disagreements and reach consensus, the 2 independent coders compare their applied codes, discuss the rationale for the codes, and address the discrepancy by either agreeing on the final application of codes or asking a third member of the research team to review. With online tools, reconciliation can take place in real-time to support collaboration and integration of multiple perspectives.

#### Step 5: Development of Findings (eg, Themes or Descriptions)

##### Content Analysis

After the coding process is complete, MyVoice teams often summarize codes with frequencies to determine the prevalence of codes and categories (ie, groups of related codes) across participants. Teams will often present code frequencies (or the proportion of responses that are aligned with a code) in their findings. Presentations of frequencies are often grouped by question or overarching main ideas across the dataset, supported by sample responses. For example, Schmid et al [41] examined the impact of COVID-19 on weight and shape control behaviors among youth. The authors conducted a content analysis that involved developing initial themes, constructing a structured codebook with codes and definitions, independently applying codes to responses for each survey question, comparing discrepancies between each pair of coders, and finalizing themes and subthemes that summarized the main ideas in responses to each question. Content analysis of the prompt, “Since the pandemic, have you changed your eating or exercise habits because you were worried about your weight or shape. Tell us about it,” resulted in 2 themes: changes for weight or shape control and changes in overall attitude or concerns. Overall, 4 subthemes that detail the responses common in each theme were presented alongside the frequency of responses. By capturing the frequencies in responses, the authors concluded that one-third of the sample implemented weight or shape control behaviors during the COVID-19 pandemic.

##### Thematic Analysis

Theme development occurs by looking for relationships across codes, both within and across questions in the dataset. MyVoice teams often develop themes through iterative team discussion where codes are grouped into related categories or concepts, patterns are identified across categories or concepts, and variation in participant experiences is described. For example, a MyVoice study examined youth perspectives on use and access to primary care [42]. Questions included:

1. Where do you go for a health problem you can’t solve on your own (family doctor, ER, urgent care, school clinic, etc)? Why do you go there?
2. Do you have a regular doctor you go to for check-ups or when you feel sick? If you don’t have a regular doctor, why not?
3. Do you think it’s important to have a regular doctor you go to for check-ups or when you feel sick? Why or why not?
4. How could your doctor’s office make your visits easier?
5. Have you had a bad experience with a doctor or clinic visit? What happened?

Using a thematic analysis approach, the research team followed the analysis steps outlined above, including data familiarization, codebook development, line-by-line coding, code reconciliation, and consolidation of conceptually related categories. To develop themes, the team held many discussions where 6 faculty and program staff reflected on patterns in the data. They considered prevalent categories (eg, convenience) in addition to those that were salient but less prevalent (eg, experiences with physical and emotional harm in health care settings). One prominent theme was “Feeling respected, valued, and heard was essential to youth having positive experiences in a primary care health care setting.” In the description of this theme and in the selection of representative quotes in the final manuscript, the authors presented the range of experiences (ie, both positive and negative previous experiences with primary care) among MyVoice participants to fully elaborate on the breadth of responses [42].

#### Quantitative Analysis

Occasionally, teams will pursue a quantitative or mixed methods approach that incorporates statistical analyses, in addition to the methods described above [43,44]. Quantitative analysis of large-scale qualitative data may be tempting because of the volume of data and the need to efficiently summarize responses. In addition, the use of spreadsheets for coding facilitates frequency counts and other descriptive methods that are less common in many approaches to qualitative research [35,38].

Several considerations should be made when performing quantitative analysis of qualitative data. First, the goal of qualitative research is typically not to generalize to a population. Decisions in the study design, including sampling, data collection, and data analysis, will likely impose limitations for a quantitative analysis. SMS text messaging responses in MyVoice are limited in that follow-up and clarifying questions cannot be asked. Therefore, analysis is restricted to the words participants choose to share. When questions are framed as very broad and open-ended, participants may only share some of their perspectives. Researchers cannot be sure that participants would not have endorsed a theme, just that they did not share it in their written responses, which creates potential missing data. As a result, quantitative analyses should align with the scope of the research question and poll question.

For example, Schuiteman et al [44] investigated self-management of health care among youth in a mixed methods study. The four MyVoice poll questions were:

1. Who schedules your doctor's appointments? Why?
2. Who usually goes with you to your doctor's appointments? Why?
3. Who usually picks up your prescription medicine? Why?
4. Do you wish you were more or less involved in your health care? How?

The primary outcome was whether respondents desired to be more, less, or equally involved in their health care (ie, responses to the fourth question), and “independence scores” were calculated to assess variation in this outcome by summing participants’ levels of independence in the first 3 questions (ie, through identification of self or help from others). Differences in the primary outcome were compared by demographic characteristics using chi-square tests. In this example, questions were worded in a way that supported coding of responses into categorical variables. Qualitative analyses explored the “why” and “how” narrative responses provided across questions, and resulting themes were compared between participants with high independence and low independence scores.

Second, careful consideration should also be given to what differences by demographics represent, particularly demographic characteristics that are socially constructed [45]. Analyses should have sufficient justification in the literature or existing theory, particularly in cases where testing for differences by demographic characteristics is desired. For example, one could expect, based on the literature, that nonbinary and transgender youth have more negative experiences with health care providers than cisgender youth [46]. In the MyVoice study of youth perspectives on primary care use and access, the research team conducted exploratory quantitative analyses to compare endorsement of each theme by different demographic groups using chi-square (for gender, race, and income level) and *t* tests (1-tailed; for age). In the study sample, more than one-third (284/789) reported previous bad experiences in a healthcare setting. Among these participants, 47% of nonbinary or transgender participants and 44% of cisgender women reported negative experiences with health care providers compared to 24% of men, which was statistically significant (*P*<.001) [42].

## Discussion

Although technology has enabled the data collection and analysis of larger volumes of text data, there has been limited practical guidance on how researchers can manage large databases and rigorously analyze large datasets using qualitative methods [3,5]. In this tutorial, we presented steps and tools we have used for team-based analysis of large-scale text data collected through a nationwide SMS text messaging poll of youth. These steps have been used by teams that include youth and trainees of all levels, supporting the development of research interest, experiences, and skills through meaningful and authentic participation in research. Novice researchers can apply the methods described here using familiar tools, like spreadsheets, to reduce the time and resources spent using other qualitative data software tools. In addition to open-ended survey responses, the data analysis methods described can be applied by other digital and mobile health researchers conducting qualitative studies with substantial samples of short text data.

This tutorial describes the application of a qualitative analysis approach to short text responses, like those in SMS text messaging, open-ended survey questions, social media posts, and more. SMS text messaging polls allow youth to share their perspectives using an easy, familiar, and accessible tool, which may support youth to feel more comfortable engaging in research [47,48]. However, responses can vary in length, detail, and quality. Unlike some other qualitative data collection methods, like semistructured interviews, MyVoice SMS text message polls are limited to a predetermined set of questions. Teams are unable to ask follow-up questions or probe for more detail, which likely limits some of the richness of the data and therefore understanding of participant perspectives and experiences [49]. Indeed, Tran et al [50] found that significantly more responses were needed in open-ended survey responses to reach the point of data sufficiency than is commonly recommended in qualitative analysis of interviews or focus groups. As a result, studies using short text responses can require larger datasets that can apply the team-based approach detailed in this tutorial. Further research is needed to illustrate analysis methods for large-scale qualitative research with longer text responses, such as interviews or observations.

Our initial study protocol proposed the use of natural language processing to support analysis of SMS text message responses. We used natural language processing to analyze several datasets and to try to streamline research processes for novice researchers [4,18]. However, time and resource limitations (eg, sufficient expertise among team members and updating algorithms for the context of each unique dataset) and concerns about the utility and validity of outputs restricted our regular use of this approach to date. Advancements in AI, including large language models and tools within qualitative data analysis software, will likely improve the efficiency and validity of AI-assisted qualitative analysis over time.

Our team includes youth as active team members in the research process to enhance the validity and relevance of the research [29,51,52]. We have found that line-by-line coding and pattern identification are processes and tools that are familiar to youth and similar to strategies taught in classrooms, enabling youth researchers to quickly engage in meaningful research opportunities. Through the MYHealth research training program, over 150 high school students and recent high school graduates have successfully used the methods in this tutorial to understand youth perspectives on future planning, social media, academic expectations, health education, and health care access [25,53]. In addition, novice researchers and researchers new to qualitative methods have successfully used our team-based approach to collaboratively and efficiently analyze large text datasets on a variety of health and health policy topics.

Despite the recent proliferation of AI tools, manual analysis procedures are still preferred in many qualitative and mixed methods studies to ensure understanding of the nuance and context available through qualitative data and limiting the bias introduced by AI that can influence findings [16,18]. This tutorial has provided an example of using a team-based qualitative analysis approach to analyze a large corpus of data collected through SMS text messaging polls of youth with commonly available tools. This tutorial adds to the limited research describing the step-by-step application of qualitative analysis techniques to large-scale qualitative data, particularly short text responses. Our approach can be used by research teams seeking to analyze short response text data using qualitative methods.

## Appendix

Multimedia Appendix 1 Recruitment, Screening and Enrollment Process.

## Abbreviations

AI: artificial intelligence
REDCap: Research Electronic Data Capture

## Data Availability

Data sharing is not applicable to this article as no data sets were generated or analyzed during this study.
