# Supplementary material for: Team-Based Analysis of Large-Scale Qualitative Data: Tutorial Using a Nationwide SMS Text Messaging Poll of Youth
Source: J Med Internet Res. 2026 Feb 27;28:e72526. doi: 10.2196/72526 (PMC12988353; doi:10.2196/72526)
Supplement: Multimedia Appendix 1 [file jmir_v28i1e72526_app1.docx]

### Recruitment, Screening and Enrollment

Youth must be between the ages of 14 and 24 upon enrollment, able to read and write in English, and use a text messaging-capable phone (from any carrier and with any operating system). During and post-COVID, recruitment shifted more toward social media and away from in-person community recruitment. Thus far, advertisements on Facebook and Instagram have been used for recruitment. Advertisements are tailored to recruit participants from particular demographic groups based on national benchmarks from the American Community Survey. At enrollment, once consent/assent is complete, participants are prompted to complete a 15-question demographic survey including measures for age, gender, race and ethnicity, zip code, highest level of education and parent’s education, and an age-appropriate validated measure of socioeconomic status (see Supplementary Material for the complete survey). Previously enrolled participants are given the opportunity to update demographic information annually by completing a survey link sent to them via text message.

Both newly recruited participants and existing participants are asked to opt-in at the start of each data collection phase. Validation for new participants occurs in a 3-step process: 1) completion of CAPTCHA during online enrollment, 2) removal of duplicate individuals by the study team, and 3) validation of personal data across platforms (online vs. text). All new participants who have completed the online consent and demographic survey are screened for duplicate IP addresses and phone numbers. Each participant is only permitted to join the MyVoice study as one individual and duplicate enrollments are removed during this step. All participants are then prompted via text message to confirm personal information provided during enrollment online and those who do not respond or fail to verify their identity are removed. Each new participant is then assigned an anonymous study ID which is used to track their progress throughout each phase. Individuals who have participated in a previous phase are also required to confirm continued participation at the start of each new phase of questions.

| *Table 1. Example demographic questionnaire completed upon enrollment* | | |
| --- | --- | --- |
| Construct/Measure | Item | Response Options |
| Date of birth | What is your date of birth? Please enter it in the following format mm/dd/yyyy |  |
| Gender | Which most closely describes your gender? | a) Woman/female  b) Man/male  c) Transgender woman/trans female  d) Transgender man/trans male  e) Nonbinary/genderqueer  f) Other (please self-describe) |
| Race | What is your race? Check all that apply. | a) Asian or Asian American  b) Black or African American  c) Indigenous American or Alaska Native  d) Middle Eastern or North African  e) Native Hawaiian or Other Pacific Islander  f) White or Caucasian  g) Other (please self-describe) |
| Hispanic/Latino Ethnicity | Are you of Hispanic, Latinx, or Spanish origin? | a) Yes  b) No |
| Zip code | What zip code do you live in? |  |
| Rurality | What would you consider the place where you live? | a) Urban  b) Suburban  c) Rural |
| Education | What is the highest level of education you have achieved? | a) 8^th^ grade or less  b) Some high school  c) High school graduate  d) Some vocational/technical training  e) Completed vocational/technical training  f) Some college  g) Completed an associate’s degree  h) Completed a bachelor’s degree  i) Some graduate school  j) Completed a master’s degree  k) Some graduate training beyond a master’s degree  l) Completed a doctoral or professional degree (PhD, JD, MD) |
| Parent’s education | What is the highest level of education any parent/guardian has achieved? | a) 8^th^ grade or less  b) Some high school  c) High school graduate  d) Some vocational/technical training  e) Completed vocational/technical training  f) Some college  g) Completed an associate’s degree  h) Completed a bachelor’s degree  i) Some graduate school  j) Completed a master’s degree  k) Some graduate training beyond a master’s degree  l) Completed a doctoral or professional degree (PhD, JD, MD) |
| Socioeconomic status | Considering your own income and the income from any other people who help you, how would you describe your overall personal financial situation? | (1) Live comfortably  (2) Meet needs with a little left  (3) Just meet basic expenses  (4) Don’t meet basic expenses |
| Socioeconomic status | Think about your family when you were growing up, from birth to age 16. Would you say your family during that time was pretty well off financially, about average, or poor? (Williams et al., 2017) | (1) Pretty well off financially  (2) About average  (3) Poor  (4) It varied |
| Income level | What is your annual household income (include parents and/or spouse, if you live with them or they support you financially)? |  |
| Income level (with above) | How many people are in your household (include parents and siblings or spouse and kids, if applicable)? | a) 1  b) 2  c) 3  d) 4  e) 5  f) 6  g) 7  h) 8  i) 9  j) 10+ |
|  | Thinking about the house you live in at the moment, do you or your parents own it or rent it? | a) My parent/guardian owns it  b) My parent/guardian rents it  c) I own it  d) I rent it  e) I don’t know |
|  | Do you have a vehicle at home (car, van, truck, etc.)? | a) Yes, one car  b) Yes, more than one car  c) No, we don’t own a car |
|  | Do you, or your parents if you live at home, receive Supplemental Nutrition Assistance Program (SNAP) benefits (e.g., food stamps)? | a) Yes, currently  b) Yes, previously  c) No, never |
